# Statistical learning of unbalanced exclusive-or temporal sequences in humans

**DOI:** 10.1371/journal.pone.0246826

**Published:** 2021-02-16

**Authors:** Laura Lazartigues, Fabien Mathy, Frédéric Lavigne

**Affiliations:** Department of Psychology, Université Côte d’Azur, CNRS, BCL, Nice, France; ICREA, University of Barcelona, SPAIN

## Abstract

A pervasive issue in statistical learning has been to determine the parameters of regularity extraction. Our hypothesis was that the extraction of transitional probabilities can prevail over frequency if the task involves prediction. Participants were exposed to four repeated sequences of three stimuli (*XYZ*) with each stimulus corresponding to the position of a red dot on a touch screen that participants were required to touch sequentially. The temporal and spatial structure of the positions corresponded to a serial version of the exclusive-or (XOR) that allowed testing of the respective effect of frequency and first- and second-order transitional probabilities. The XOR allowed the first-order transitional probability to vary while being not completely related to frequency and to vary while the second-order transitional probability was fixed (*p*(*Z*|*X*, *Y*) = 1). The findings show that first-order transitional probability prevails over frequency to predict the second stimulus from the first and that it also influences the prediction of the third item despite the presence of second-order transitional probability that could have offered a certain prediction of the third item. These results are particularly informative in light of statistical learning models.

## Introduction

Adapting behavior to complex environments relies on the ability of the cognitive system to capture and learn relations between stimuli linked by statistical distributions [1–5]. Statistical learning can depend on both explicit and implicit processes [6, 7], and it can be observed in animals, including nonhuman primates [8, 9] and rats [10, 11]. Statistical learning can occur in different sensory modalities such as auditory [12], visual [13] and tactile [14]. This type of learning has also been shown to occur in humans early in life, for example, to account for word segmentation [15–18]. Considering that the cognitive system can use transitional probabilities between adjacent elements to extract segments, statistical learning can appear to be a rudimentary learning process. Nevertheless, many refined aspects of this learning process are still poorly understood. For instance, a pervasive issue in statistical learning has been to determine the parameters that sustain regularity extraction, such as conditional probabilities, frequency, and their reciprocal influence [19]. Regarding learning of temporal stimuli, there are essentially two crucial parameters that describe a sequence of stimuli: the frequency of the sequence and the transitional probability of an item in the sequence given the preceding one(s). The ability to detect serial patterns requires estimation i of the frequency of an item or a group of items (also known as distributional learning, [20]) and ii the transitional probability of an upcoming item given the preceding one(s) (also known as conditional probabilities, [21]). The frequency of a sequence of two items, such as (*X*, *Y*), corresponds to the number of occurrences of the sequence *XY* among a set of sequences. The transitional probability (TP) *p*(*Y*|*X*) corresponds to the number of occurrences of *Y* given *X*. The TP corresponds to the strength with which *Y* can be predicted by *X* in a sequence of events [22].

Both frequencies and transitional probabilities can be fully learned based on the statistical relation between a series of events embedded in a set of sequences. This raises the issue of determining which type of information is predominantly learned. On the one hand, if learning is aimed at detecting a stimulus or a given sequence among others, frequency should prevail, as is the case for Hick’s Law, according to which responses to more frequent stimuli are faster [23]. On the other hand, if learning is involved in predicting future events within sequences, then the transitional probability of an item given a preceding one should be a more relevant source of information.

Until now, the TP index has been considered the most fundamental process in statistical learning [24], and it has been used extensively to track how adjacent and non-adjacent elements are processed in diverse domains (e.g., artificial grammar learning, [12, 25]; ASRT tasks, [26]). This notion has, for instance, proved crucial to describe how individuals acquire language [27, 28], how visual patterns can be captured [29], how sequences can be anticipated regarding preceding actions [30], and how semantic predictions develop based on learned associations [31]. The learning of TPs involves patterns that can occur in different sequences, with the consequence that the TP of an item given a preceding one (*p*(*Y*|*X*)) can be different from the frequency of the *XY* sequence. The reason is that the frequency of all sequences embedding *X* and *Y* need to be taken into account globally [32, 33]. Indeed, if an item *X* occurs in several sequences (e.g., *XY* and *XT*), the frequency of the sequence *XY* does not correspond to the TP *p*(*Y*|*X*). Therefore, the TP in a pair depends on the frequency of its constituent in all of the encountered sequences taken together. For instance, when sequences are made of three items, the TP *p*(*Y*|*X*) not only depends on the frequency of all triplets in which the pair *XY* occurs (*XYZ*, *XYT*, etc.), but also on all the triplets in which one of the items of the pair occurs (*XWT*, etc.). A consequence is that although the frequency of all sequences containing *X* and/or *Y* determines TP, which seems computationally demanding, TP could remain the most relevant information in sequence learning. Given that for a given *XY* pair of adjacent items the frequency and TP can have different values, the related question is then to what extent the learning of TP can be biased by the frequency of the sequence of items.

For example, considering a restaurant proposing three course menus including ‘Salad’, ‘Dish’, ‘Dessert’, the relevant information for the cognitive system to predict events should be the TP between the different meals. We choose this specific example because anyone can have a good grasp of the ‘restaurant script’ as a sequence of expected behaviors [34], and as such it is easy to keep in mind the order of the three meals to follow explanations. Note that the prediction of the sequences can appear trivial for the reader, but just because anyone can almost perfectly integrate the plausibility of the successive events. To play around with some numbers a bit more precisely, let us imagine that one fifth of the menus are Salad-Dish, one fifth Salad-Dish-Dessert, and three fifths are Dish-Dessert. In that case the frequency of Salad-Dish is lower (two fifths of all sequences) than the frequency of Dish-Dessert (four fifths of all sequences). However, the TP varies in the opposite direction to frequency, with *p*(*Dish*|*Salad*) = 1 (a dish always follows a salad) and *p*(*Dessert*|*Dish*) = .80 (dessert follows a dish four times out of five). Considering frequency and TP separately allows one to test if the learner focuses more on frequency than on TP. Frequency and TPs are known to be both involved in different types of learning, in particular for the extraction of words which depends on both distributional statistical structure and conditional statistical structure [19]. For instance, frequency, such as the frequency of a word can be relevant to help categorizing a word as a content word or a function word [35]. Also, a high frequency can facilitate the acquisition of a word in comparison to a rare item [36]. On the other hand, TPs are rather involved in the encoding of associations based on the predictability of an element as a function of a preceding one [37], and can be very informative when, for instance, two words are very frequent but the transitional probability between the two is low. In our example of the restaurant, we can consider two different situations where someone must prepare for the future. First, the owner should be sure to have enough ingredients to serve all customers but not more than necessary to avoid waste. He would then focus on the frequency of the different meals ordered by consumers to buy the right amounts of ingredients. Second, the cook should optimize his time to prepare dishes in order to serve customers on time. He would then need to focus on the TP between the different meals to prepare for the next course, as a function of the preceding meals ordered by the client. For instance, the TP *p*(*Dish*|*Salad*) = 1 should override the low frequency of the pair *f*(*Salad* − *Dish*) = .40 to predict the upcoming meal once the Salad is being served. By analogy, an experimental design manipulating temporal sequences allows prediction of items as a function of the preceding ones, and should then lead participants to use TPs.

The preceding example shows that processing of sequences of more than two items involves processing several pairs of items. This implies that in sequences of more than two events, such as triplet *XYZ*, there are two different kinds of TP: first-order TP (i.e., *p*(*Y*|*X*) or *p*(*Z*|*Y*) in a sequence XYZ) and second-order TP (i.e., *p*(*Z*|*XY*)), which may tap on different learning processes [38–40].

In our example, the second-order TP *p*(*Dessert*|*Salad* − *Dish*) = .5 because after a salad and a dish, customers choose a dessert half the time. However, when the customer orders only a dish, the owner will expect a dessert to be ordered every time (*p*(*Dessert*|*NoSalad* − *Dish*) = 1). Therefore, the second-order TP brings more certainty to the prediction of dessert (*p*(*Dessert*|*NoSalad* − *Dish*) = 1) than the first-order TP (*p*(*Dessert*|*Dish*) = .80). The optimal prediction of the third item of a sequence should then use second-order TP instead of first-order TP. However, although second-order is sufficient to exactly predict the third item in a sequence, it requires combining three items, which is more resource demanding than the learning of first-order TP by combining only two items.

Regarding our example, let’s imagine four types of customer profiles: the first is on a diet and never orders dessert (*Salad* − *Dish* − *NoDessert*); the second is a big eater and orders a dish and a dessert (*NoSalad* − *Dish* − *Dessert*); the third is a small eater and orders a salad and a dessert (*Salad* − *NoDish* − *Dessert*); the last one is the unwelcome customer who does not order anything from the menu (i.e., *NoSalad* − *NoDish* − *NoDessert*) except a cup of coffee. In this case, all first-order TP are equal to.5 and all second-order TP are equal to 1. Presuming that the four types of customers have equal frequencies, the four sequences make a typical ‘Exclusive Or’, or XOR for short (e.g., Salad OR Dish, but not both, means that the client is willing to order a Dessert). Our manipulation of the frequency of the four triplets within the XOR allows a dissociation between first-order TP and frequency and to vary first-order TP for fixed values of second-order TP. Let us imagine a sample of twenty customers, among them eight dieters (*Salad* − *Dish* − *NoDessert*), five big eaters (*NoSalad* − *Dish* − *Dessert*), five small eaters (*Salad* − *NoDish* − *Dessert*), and two bad customers (*NoSalad* − *NoDish* − *NoDessert*). In that case, all second-order TP are *p*(*Dessert*|*Salad* − *Dish*) = 1, while first-order TP and frequency are partly dissociated, as *p*(*Dish*|*NoSalad*) = 5/7 = .71 has the highest TP, while *f*(*Salad* − *Dish*) = 8/20 = .4 is the most frequent pair.

A few previous studies have addressed the issue of the combined effects of TPs and frequency during statistical learning of three-items sequences. For instance, the study by Mirman, Graf Estes and Magnuson (2010) on statistical learning of an artificial grammar by the simple recurrent network (SRN) model [41] showed an advantage for the frequency at the beginning of training, followed by a progressive advantage for TP as learning progressed. Their artificial grammar comprised twelve syllables that were used to create four pseudowords (called words in their study). Two words were presented 400 times (high frequency words), while two other words were presented only 200 times (low frequency words). The words were presented randomly in a continuous stream, so the high frequency words created part-words still with a higher frequency than the low frequency words. With this setting, first-order TPs were all equal to 1 for words (no matter their frequency), but only.58 for part-words. Their results showed that high frequency words were quickly learned by the model, but part-words still had an advantage in the beginning of the learning process over low frequency words. With learning, this pattern tended to reverse, with the model showing better learning of low frequency words compared to part-words. Their results suggested that frequency is initially used for statistical learning, but that the extraction of TPs progressively emerges during learning up to a point where they become prevalent.

Another study by Endress and Langus (2017) used sequences of three visual items to test the respective role of frequency and TPs in memorization. After a familiarization phase consisting of a stream of sequences, participants had to choose between a high TP sequence that appeared in the familiarization phase (called words in their study, for instance ABC or DBF), a low TP sequence that appeared in the familiarization phase by the contact between two words (called part-words, for instance FAB corresponding to the contact between the words ABC and DBF), and a high TP sequence that never appeared during the familiarization phase (called phantom word, for instance ABF, built from the two frequent pairs AB and BF present in the familiarization phase, although their combination never occured). Their results showed that words were preferred to part-words but not to phantom words. This suggested that participants were successful in identifying words versus part words based on their TPs difference, but failed to identify words versus phantom words based on their frequency difference. Also, phantom words were preferred to part-words when the stream during the familiarization phase was continuous. When the words were separated by a blank in the familiarization phase, then a preference for words over phantom words appeared. This suggested that participants can identify words versus phantom words on the basis of frequency, but they need more clues. Thus, these results suggested a greater impact of TPs compared to frequency. However the authors noted that participants could reject part-words more easily because they did not have the correct elements at the boundary of the sequence. For example, if the words were ABC and DBF the part-word BCD did not correspond to the edge of words, but the phantom words ABF did and this effect could partly explain the results of their experiment. Note that other studies that have used this protocol with auditory syllable sequences found variable effects depending on the native language of the participants [42, 43], but the study of Endress and Langus (2017) based on visual items let more clearly reveal a preference for TPs over frequency. The experiments of Mirman et al. (2010) and Endress and Langus (2017) provide information on the respective effects of TPs and frequency during statistical learning of three-items sequences. However, Mirman et al. (2010) focused on modeling, not on human experimental data. Endress and Langus (2017) used a delayed test to measure recognition of previously learned sequences. However, offline measures do not allow a precise tracking of the learning process at play, in particular when sequences are composed of several successive items. The present study then targeted online measures of the prediction of the successive items within sequences to investigate then relative effects of TPs and frequency in humans.

Based on this previous literature, our main hypothesis was that in a task involving the prediction of serial events, the relevant to-be-learned information between pairs of items should be first-order TP and not frequency. Further, although the relevant to-be-learned information between triplets of items should be second-order TP, learning difficulty of second-order TP may lead to effects of the first-order TP. The aim of the present study was therefore to investigate to what extent prediction in humans predominantly involves frequency, first-order TP or second-order TP during learning. To this end, we used repeated presentation of four sequences of three items combined according to the exclusive-or (XOR) logic [32, 33, 44].

The XOR logic is a benchmark for studying the relations between learning of first-order TP, second-order TP and frequency. The to-be-learned material had two properties: items were presented sequentially to test the effect of first-order TP (having values less than one) and the effect of second-order TP (always having value of one). The highest value associated with second-order TP was an incentive. The frequency of the sequences of items involved in the XOR were also manipulated to make first-order TP vary as a function of the frequency of the sequences. To summarize, the main questions that arise are: 1. to what extent first-order TP prevails over frequency to predict the second item in a sequence and 2. to what extent prediction of the third item is optimal and uses only second-order TP. Triplets are known to be straightforward to study adjacent and nonadjacent dependency learning [25, 45, 46]. Although first-order TPs (see [25, 47]), second-order TPs (see [48]) and the interaction between first-order TPs and frequency [49, 50] have been studied, the effect of second-order TPs as a function of first-order TPs and the frequency of the sequences is still poorly understood. The possibility for frequency biasing the learning of TP makes different predictions. Optimal temporal learning should encode both the values of second-order TP (on the third item in the sequence) and the values of first-order TP (on the second item), not of the frequency of the sequences. Focusing on TP should override frequency effects. However, although the relevant information to learn the combinations is the TP, frequency is reported to have strong effects on learning [51]. It is therefore not clear if frequency of the sequences could bias the processing of TP between items, and if first-order TP could bias the processing of second-order TP.

## Materials and methods

In the present study, we used sequences of three items of the form *X* − *Y* − *Z*, with *X*, *Y* and *Z* being presented in this temporal order. This allowed us to embed both first-order transitional probabilities (*p*(*Y*|*X*), *p*(*Z*|*Y*) and *p*(*Z*|*X*)) and second-order transitional probabilities (*p*(*Z*|*XY*)) while manipulating the frequency of sequences.

The experimental design consisted of four triplets of the form *X* − *Y* − *Z* that combined items according to an XOR logic [33]. The XOR allows us to dissociate between first-order TPs (*p*(*Y*|*X*), *p*(*Z*|*Y*), and *p*(*Z*|*X*)) and second-order TPs *p*(*Z*|*XY*). Interestingly, while the third item can be fully predicted by the combination of the first two stimuli (i.e., (*p*(*Z*|*XY*) = 1), it cannot be fully predicted by one of the two preceding stimuli alone (e.g., (*p*(*Z*|*Y*)≠1) and (*p*(*Z*|*X*)≠1)). It was then possible to manipulate the frequency of the sequences and different values of first-order TP for a fixed value of second-order TP.

### Participants

Thirty-eight psychology students at Université Côte D’azur, between 18 and 30 years of age (33 females and 5 males, mean age 22.4) participated in the experiment voluntarily (21 among them received course credits for their participation). To estimate the minimal sample size to achieve a power of.80, we referred to the results of Endress and Langus (2017) who studied the effects of TPs and frequency on statistical learning of visual sequences using between 20 and 30 participants in each of their experiment. Endress and Mehler (2009) and Perruchet and Poulin-Charronnat (2012) used the same protocol with audio sequences and had respectively 14 participants per experiment [42], 40 participants for experiment 1 and 2 and 28 participants for experiment 3 [43]. Although our protocol is not entirely comparable to theirs, we expected similar effect sizes to occur. Therefore on the basis of these previous experiments, a stop criterion of forty participants was chosen (i.e., corresponding to the highest number of participants used by Perruchet and Poulin-Charronnat (2012) in their Exp. 1). In the present study, forty students completed the experiment but two of them (aged 40 and 67) were excluded because they did not correspond to our age criterion. The experiment was approved by the local ethics committee (CERNI) of the Université Côte d’Azur and the experiment was conducted with the informed written consent of the participants.

### Material

**Fig 1:**
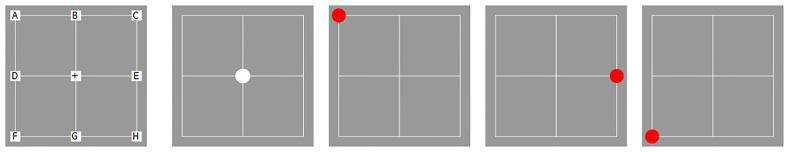
Stimulus coding according to the virtual grid of nine positions (left display), followed by successive displays exemplifying one trial (i.e., a sequence AEF).

The experiment was programmed in PsychoPy [52], and stimuli were displayed on a touchscreen. Nine positions were marked by nine crosses ‘+’ displayed on a 3 × 3 virtual grid on a gray background (Fig 1). Each sequence was made of three dot stimuli that replaced the crosses successively at different positions on the touchscreen. Each participant was presented with a set of four sequences following the XOR logic. While respecting the XOR logical structure, the presentation of the sequences was unbalanced by assigning different frequencies to the four sequences, respectively .40, .25, .25 and .10 (Table 1). Each set of four sequences was selected for each participant on the basis of her or his own response times during a training phase. During this training phase, 224 random sequences of three dot stimuli were displayed to measure all of the possible transition times between all dot positions for each participant. Based on these data, a set of four sequences was selected for each participant, so that the eight corresponding transitions times were as close as possible.

**Table 1:** Transitional probability (TP) and frequency associated with each sequence and each type of transition (*B*|*A* and *C*|*B*).

Sequence	Freq.	*B*|*A*	TP1	*C*|*B*	TP2	2*^nd^* Order TP
ABC	.40	.40/(.40 + .25) =	.62	.40/(.40 + .25) =	.62	1
AEF	.25	.25/(.40 + .25) =	.38	.25/(.10 + .25) =	.71	1
DBF	.25	.25/(.10 + .25) =	.71	.25/(.40 + .25) =	.38	1
DEC	.10	.10/(.10 + .25) =	.29	.10/(.10 + .25) =	.29	1

Notes. All second order transitional probabilities (i.e., the combination of the two first stimulus predicting the third) are equal to 1 due to the XOR logic.

### Protocol and task

The experimental phase was made of 400 trials divided into 10 blocks of 40 trials. Each trial consisted in presenting one of the 4 sequences of three dots governed by the XOR. The number of occurrences of each sequence within each block depended on its frequency (Table 1). The 400 trials allowed us to record TT1 (i.e., Transition Time 1, corresponding to the time taken to reach the second point) and TT2 (i.e., Transition Time 2, corresponding to the time taken to reach the third point) within each trial.

A trial started when the participant touched a white dot that was always displayed at the center of the screen. The white dot then disappeared and was replaced by the first red dot displayed at one of the eight remaining locations. When the participant touched the first red dot, it was instantly replaced by the second red dot at one of the seven remaining locations (i.e., Inter-Stimulus Interval (ISI) between dot stimuli were set to zero milliseconds). The second red dot was then instantly replaced by the third red dot at one of the six remaining locations. Whenever the participant touched the second or the third red dot within a sequence, the response time was recorded as TT1 and TT2, respectively.

The next trial began immediately after completion of a sequence by touching the third red dot, with the white dot being displayed again at the center of the screen. Participants were instructed to touch the red dots as quickly as possible.

At the end of the 400 trials learning phase, a switch phase was presented to test the level of learning during the learning phase. The switch phase consisted in the permutation of the third red dots between the different sequences (i.e., ABC, AEF, DBF and DEC in the learning phase became ABF, AEC, DBC and DEF during the switch phase). All pairs of stimuli in the sequences of the learning phase were preserved during the switch phase. However, the combinations of pairs within triplets were novel. For example, AB and BF were present during the learning phase, but the sequence ABF was new. The transition AB in the learning phase was preserved during the switch phase, but the sequence ABC was not. The switch allowed us to test if participants learned second-order TP according to the XOR logic, with TT2 expected to decrease during the learning phase (blocks 1 to 10) and to increase during the switch phase. As a consequence, the structure was still an XOR in the switch phase. The new triplets were built based on the exact same logical TPs between the successive dots, and the exact same pairs of dots were used between the transitions. However, the third elements were now unpredictable based on the combinations learned during the learning phase. If participants learned how the three stimuli in the sequences could logically have been combined based on second-order TPs during the learning phase, TT2 were expected to increase during the switch phase. Effectively, if learning of the XOR takes place during the learning phase, we should expect a decrease of TT2. A decrease of TT2 during the learning phase would confirm that the participants could form a reliable representation of the whole triplets and not only representations of the pairs involved in the triplets. A decrease of TT2 during the learning phase could also unfortunately indicate faster response for the third point for other reasons than learning the XOR. However, increase of TT2 during the switch would disconfirm the uniqueness of these other reasons and would confirm that learning of the XOR took place during the learning phase.

## Results

Deidentified data and analyses for Experiment 1 are posted at https://osf.io/ank3p/files/.

We first removed 4% of the data corresponding to RTs faster than 150 ms and slower than 850 ms (±2 standard deviations from the mean). RTs were analyzed by fitting linear mixed-effect models (LMM) to the data, using the lmer function from the lme4 package in R [53].

**Fig 2:**
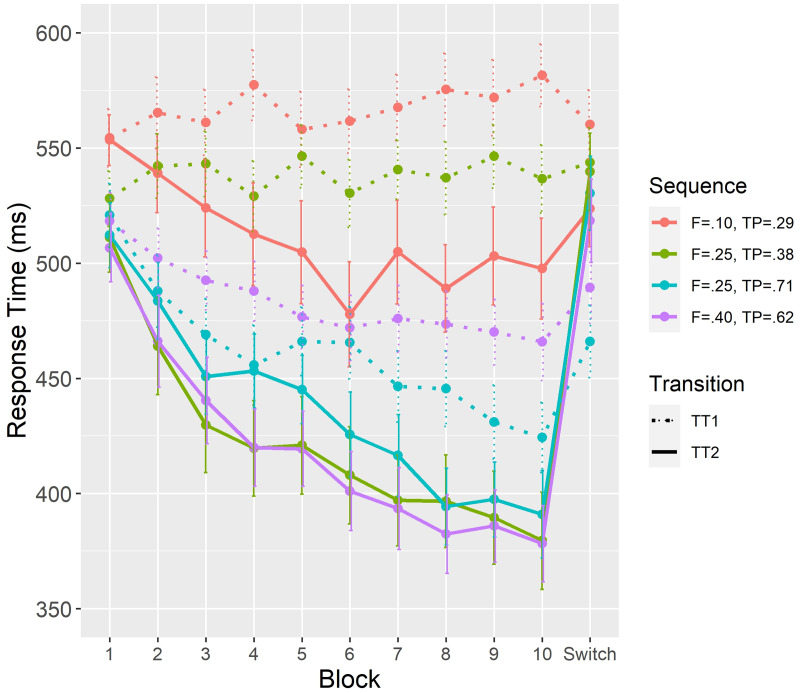
Average response times between the first and second stimulus (TT1) and between the second and third stimulus (TT2) as a function of block number, frequency and first-order TP values. Note. Second-order TPs are always equal to 1; the red lines correspond to the sequence DEC in Table 1, the purple lines to ABC, the dotted green line and the plain blue line to AEF, and the dotted blue line and the plain green line to DBF.

Fig 2 shows, for each of the four sequences, the evolution of response times (*RT*) to reach the second position (TT1) and the third position (TT2). TTs are reported for each block of the learning phase (10 first blocks) and for the switch phase (last block). Analyses were carried out to test for the effect of first-order transitional probability and type of transition (TT1 vs TT2) for the learning phase (block 1 to 10), but for the switch phase (block 11), only TT2 has been taken into account because the modifications created by the switch were related to the third stimulus.

### Effects of TPs on TTs

A global analysis indicated a significant effect of transition type on RTs, with TT2 being shorter than TT1 (*F*(1,32104) = 1897.5, *p* <.001). This suggests that the type of TP (first-order vs second-order) accounts for the value of TT, TT2 for second-order TP being shorter than TT1 for first-order TP because second-order TP had higher values of probability than first-order TP.

Regarding the values of first-order TP, the mixed model in Table 2 also showed that, early in the first learning block, the lowest value of first-order TP produced RTs (*RT*_*TT*1+*TT*2_) longer than the three other highest values of first-order TPs (*F*(3,2905) = 19.8, *p* <.001). To investigate more precisely how first-order TP and second-order TP were learned, the following analyses separately consider TT1 (involving solely first-order TPs on the processing of the second item) and TT2 (involving a combination of first-order TPs between the second and third items and second-order TP between the first two items and the third item).

**Table 2:** Effects of transition type (TT1 vs TT2) and first-order TP on RTs in the first block.

Variable	Sum Sq.	Mean Sq	NumDF	DenDF	F Value	*p*
Transition Type	52583	52583	1	2905	7.459	.006**
First-order TP	418043	139348	3	2905	19.766	<.001***
Transition Type * First-order TP	16679	5560	3	2905	0.789	.500

### Learning of first-order TPs

Regarding *RT*_*TT*1_, the mixed model 1 (Table 3) showed a significant effect of block number on *RT*_*TT*1_, with RTs that decreased during the learning phase of the experiment (*F*(1,14650) = 153.5, *p* <.001). This result suggests that the first and second stimuli in the sequences were paired during learning, leading to progressively faster response times on the second stimulus. Considering learning as a function of the value of first-order TP, we analyzed the effect of TP values controlled for the frequency of the pairs of first and second stimuli. This was made by comparing TT1 between the two sequences AEF and DBF (cf. Table 1) that had equal frequency (dashed green line and dashed blue line, respectively, in Fig 2). Analyses in Table 4 showed a significant interaction between block number and first-order TP for these two sequences of equal frequency (*F*(1,7309) = 128.2, *p* <.001). *RT*_*TT*1_ were longer for the lowest first-order TP value than for the highest value. This result shows that the first-order TP itself had an impact on learning and processing of transitions from the first and second stimuli.

**Table 3:** Effects of block number on *RT*_*TT*1_ for the model 1 and *RT*_*TT*2_ for the model 2 during the learning phase.

Model	Variable	Sum Sq.	Mean Sq	NumDF	DenDF	F Value	*p*
1	Block Number	1728388	1728388	1	14650	153.52	<.001***
2	Block Number	17017442	17017442	1	14518	1230	.001***

**Table 4:** Effects of first-order TP and block number on *RT*_*TT*1_ during the learning phase for sequences with equal frequency.

Variable	Sum Sq.	Mean Sq	NumDF	DenDF	F Value	*p*
First-order TP	253653	253653	1	7309	24.928	<.001***
Block Number	957798	957798	1	7309	94.129	<.001***
First-order TP * Block Number	1304034	1304034	1	7309	128.155	<.001***

Notes. The studied sequences correspond to the dashed green line and the dashed blue line on Fig 2.

To further investigate whether first-order TP between the two stimuli could take precedence over frequency of the pair, we next compared the sequence DBF with the first-order TP equal to.71 and frequency of.25 (dashed blue line) and the sequence ABC with the first-order TP equal to.62 and frequency of.4 (dashed purple line). The result in Table 5 indicated an interaction between first-order TP values and block number (*F*(1,9557) = 31.8, *p* <.001). The *RT*_*TT*1_ were shorter for the highest value of first-order TP despite the lowest value of frequency (DBF, dashed blue line), compared to the first-order TP corresponding to the lowest value despite the highest value of frequency (ABC, dashed purple line). This comparison shows that TT1 depends more on first-order TPs values within the two successive stimuli than on the frequency of the pair.

**Table 5:** Effects of first-order TP and block number on *RT*_*TT*1_ during the learning phase, for sequences allowing a true separation of first-order TP and frequency.

Variable	Sum Sq.	Mean Sq	NumDF	DenDF	F Value	*p*
First-order TP	4503	4503	1	9557	0.619	.431
Block Number	3419177	3419177	1	9557	469.896	<.001***
First-order TP * Block Number	231302	231302	1	9557	31.788	<.001***

Notes. The studied sequences correspond to the dashed purple line and the dashed blue line on Fig 2.

### Learning of second-order TP

Regarding *RT*_*TT*2_, the mixed model 2 in Table 3 showed a significant decrease of *RT*_*TT*2_ as a function of block number during the learning phase (*F*(1,14514) = 600.5, *p* <.001). This effect supports the idea that learning impacted *RT*_*TT*2_. Further, *RT*_*TT*2_ significantly increased in the switch phase compared to the last block of the learning phase (*F*(1,2831) = 730.4, *p* <.001). In the switch phase, the third positions were switched between sequences to create new sequences corresponding to a new XOR, in which second-order TPs were changed (reset from 1 to zero, considering that the participant would adopt the previously learned sequences on the new sequences) but first-order TPs were not changed (see the Methods section). This result shows that second-order TPs were actually learned (and not only first-order TPs) to influence *RT*_*TT*2_. This findings indicates that learning of the second-order TP involved learning the whole sequences of three stimuli, on top of the pairs.

Although the previous analysis showed that the second-order TPs were learned, all sequences were not learned equally, even though theoretically all second-order TPs equaled 1. Indeed, the analysis in Table 6 showed a significant interaction between block number and first-order TP on *RT*_*TT*2_ (*F*(1,14514) = 20, *p* <.001). Therefore, three separate models were tested to examine the effect of frequency and first-order TP to obtain the best account of the data by computing the AIC [54] for each model (see Table 7). Lower AIC corresponds to a more optimal model (i.e., a more parsimonious model). The results showed that the model including first-order TP and block number was better than the model including frequency and block number. Moreover, the model including all factors was not better than the model including only first-order TP and block number. Fig 2 shows that the three sequences with the highest values of first-order TP (TP = .38, TP = .62 and TP = .71) exhibited the same learning curve without significant difference (*F*(2,13084) = 1.06, *p* = .347), but the sequence with the lowest value of first-order TP (TP = .29, plain red line) exhibited the longest *RT*_*TT*2_ compared to the other three sequences (*F*(1,7243) = 43.64, *p* <.001). For this sequence, *RT*_*TT*2_ did not increase between the last block of the learning phase and the switch phase (*F*(1,251) = 2.9, *p* <.09). This result suggests that a too low value of first-order TP might preclude processing of second-order TP.

**Table 6:** Effects of block number and first-order TP on *RT*_*TT*2_ during the learning phase.

Variable	Sum Sq.	Mean Sq	NumDF	DenDF	F Value	*p*
Block Number	10967459	10967459	1	14512	836.210	<.001***
First-order TP	815174	271725	3	14512	20.718	<.001***
Block Number * First-order TP	546698	182233	3	14512	13.894	<.001***

**Table 7:** Comparisons of mixed-model based on the AIC for the learning phase of *RT*_*TT*2_.

Model	AIC
Block * Frequency	179 559.5
Block * First-order TP	179 528.1
Block * Frequency * First-order TP	179 528.1

## Discussion

The purpose of this study was to investigate learning of first-order and second-order transitional probabilities (TP) in temporal sequences and their modulation by frequency. The novelty of our approach is to cross vary the values of first-order TP and the values of frequency of the sequences and to vary the values of first-order TP for fixed values of second-order TP in the learning of temporal triplets. This was done with the experimental design based on the Exclusive Or (XOR) that allowed statistical learning of both TP and frequency. Our hypothesis was that the temporal feature of the sequences would prompt participants to favor TP over frequency, because TP allows one to predict the next item of a sequence whereas the frequency does not.

### First-order TP

A first set of results linked to *RT*_*TT*1_ confirm the hypothesis that first-order TP prevails over frequency when learning a pair of first and second items in a triplet. The decreasing values of *RT*_*TT*1_ with learning blocks were inversely proportional to the values of first-order TP, but not to the values of frequency. This sensitivity of *RT*_*TT*1_ to first-order TP indicates that the second stimulus (e.g., B) can be paired to a preceding one (e.g., A) in a forward manner, instead of being simply represented as an unordered pair. This prevalence of first-order TP over frequency in the prediction of temporal events is in accordance, for instance, with the study by Mirman, Graf Estes and Magnuson (2010). Note that in the present study, exact values of first-order TP could be learned early in the first learning block, making frequency less useful in the next learning blocks of the experiment. However, note that in the study by Mirman, Graf Estes and Magnuson (2010), the manipulation of TP was stronger than the manipulation of frequency: the TPs in their words were equal to 1 and the TPs in their part-words were equal to.58, but the difference in frequency between low frequency words and part-words was weak (200 presentations per cycle for low frequency words and 229 or 241 presentations per cycle for part-words, which is 17.5% more frequent than the low frequency words) in comparison to our study. In the present study, the difference in frequency and the difference in first-order TP across sequences were equally important, and we believe that our setting allowed us to better test the relative effect of these two factors. Nevertheless, our finding is that first-order TPs prevail over frequency to learn the first pair of a triplet. This finding confirms the results of Endress and Langus (2017) but more crucially, we believe that our method completes theirs. Effectively, Endress and Langus (2017) focused on an offline measurement based on recognition after a familiarization phase, which helped study the effect of TPs and frequency on memorization. In contrast, the present study used an online measurement of response times within sequences allowing us to observe in real time the effect of TPs and frequency on the prediction of each item in a sequence. Moreover the question of memorization is assessed by the presence of the switch phase allowing to test the learning of each sequence. Also, note that Endress and Langus (2017) discussed a possible edge effect in their study, that could have allowed to better reject part-words compared to words and phantom words, hence biasing their results in favor of TPs. The present study however allows us to conclude that first-order TPs can prevail over frequency in the absence of edge effects, since each of our sequences began and ended with similar items.

### Second-order TP

Our second finding showed a decrease of *RT*_*TT*2_ for the second transition, indicating that the third stimulus was associated with the combination of the two preceding stimuli in the sequences. Further, the increased *RT*_*TT*2_ in the switch phase compared to the last learning block showed that second-order TPs were actually learned by participants. Indeed, the switch was implemented to reset the second-order TP values to zero during the switch. Along with learning curves steeper for *RT*_*TT*2_ than for *RT*_*TT*1_, this result shows a capacity to combine stimuli as complex triplets (i.e., not only represented as successive groups of pairs). The design based on the XOR therefore appears useful to show that learning of sequences goes beyond the ability to represent pairs of items based solely on first-order TP.

Finally, the three sequences with the highest values of frequency and first-order TP did not produce significant differences on *RT*_*TT*2_, while the lowest value of frequency and first-order TP impaired processing of the second-order TP. The AIC show that this effect is mainly based on the first-order TP and not on the frequency. This result suggests that second-order TP can be extracted to serve the prediction of the third item under the condition that first-order TP is high enough. Although second-order TP is necessary and sufficient for optimal prediction of the third item, our findings tend to show that both first-order TP and second-order TP influence the transition times to the third item. If first-order TP is too low, then second-order TP is not sufficient to process the third item.

The present findings showed a prevalence of first-order TP over frequency, and the extraction by participants of both first-order TP and second-order TP. These findings confirm our hypothesis that the most relevant information for learning temporal sequences is the transitional probability. This argues for the functional role of TP in order to predict successive elements in temporal series, in accordance with previous observations [22, 41]. Our design based on the XOR improves our understanding of the role of TP by showing that participants can use second-order TP based on the combination of the two preceding items. This ability to extract the combination of two preceding items to better predict the following one takes the form of a quite complex computational representation of probability values. Such learning of second-order TP challenges our understanding of combinatorial learning [32, 33]. Biologically inspired models of learning encode transitional probability through the efficacy of synapses [55, 56]. When the synaptic dynamics follow a Hebbian type of modification as a function of the temporal contiguity between events, synaptic efficacy can encode first-order transitional probability (see [57]). Assuming a simple Hebbian learning, synapses between neurons coding for A and neurons coding for B have been reported to potentiate when the two pre- and postsynaptic neurons are active, which is realized when the sequence AB is encoded. These same synapses between A and B would depress when one neuron A or B only is active, which is the case when encoding sequences AE or DB. Such encoding of first-order TP allows populations of neurons coding for the items in memory to activate each other in sequences, allowing the reproduction of learned sequences (see [31, 56, 58]). The learning of first-order TP by synapses indicates that temporal contiguity is a fundamental property of events to be encoded in memory. However, standard synaptic learning mechanisms do not account yet for learning of second-order TP, such as those reported in the present study (see [32] for a discussion). Combinatorial learning of second-order TP is made possible thanks to additional learning mechanisms involving mixed-coding neurons [59–63] or a generalized Hebb rule of intersynaptic learning [32, 33]. The later studies have shown that the synergy of these mechanisms allows reproduction of sequences of three items embedding XOR combinations [32, 33]. These later models encode first and second-order TP in synapses as a function of the frequency of the sequences. We then expect that the present study can provide valuable behavioral data on the way different values of frequency lead to synaptic learning of different values of first and second-order TP. Behavioral data can then constrain model parameters to allow models to reproduce a larger variety of behaviors related to learning and prediction in temporal sequences.

In the case of complex combinatorial learning of pairs or triplets of items, learning could be more robust in unbalanced cases, that is when the first-order TP is not evenly distributed among sequences [64], as in the present study. By manipulating the frequency of the sequences, the higher values of first-order TP could, for instance, be learned more rapidly while lower values of first-order TP could be neglected or learned with more difficulty [26].

## Supporting information

S1 File (TXT)
